# A Portable Dynamic Laser Speckle System for Sensing Long-Term Changes Caused by Treatments in Painting Conservation

**DOI:** 10.3390/s18010190

**Published:** 2018-01-11

**Authors:** Alberto J. Pérez, Rolando J. González-Peña, Roberto Braga, Ángel Perles, Eva Pérez–Marín, Fernando J. García-Diego

**Affiliations:** 1 Dep. d’Informàtica de Sistemes i Computadors (DISCA), Universitat Politècnica de València, 46022 Valencia, Spain; aperez@disca.upv.es (A.J.P.); aperles@disca.upv.es (A.P.); 2 Dep. Fisiología. Unidad de Biofísica y Física Médica. Facultad de Medicina y Odontología, Universitat de València, 46010 Valencia, Spain; 3 Dep. Engenharia (DEG), Universidade Federal de Lavras (UFLA), 3037 Lavras, Brazil; robbraga@deg.ufla.br; 4 ITACA Institute, Camino de Vera, s/n, 46022 Valencia, Spain; 5 Dep. de Conservación y Restauración de Bienes Culturales, Universitat Politècnica de València, 46022 Valencia, Spain; evpema@crbc.upv.es; 6 Dep. de Física Aplicada, Universitat Politècnica de València, 46022 Valencia, Spain; fjgarcid@upv.es

**Keywords:** dynamic speckle, activity, temporal history speckle pattern, varnish, cyclododecane

## Abstract

Dynamic laser speckle (DLS) is used as a reliable sensor of activity for all types of materials. Traditional applications are based on high-rate captures (usually greater than 10 frames-per-second, fps). Even for drying processes in conservation treatments, where there is a high level of activity in the first moments after the application and slower activity after some minutes or hours, the process is based on the acquisition of images at a time rate that is the same in moments of high and low activity. In this work, we present an alternative approach to track the drying process of protective layers and other painting conservation processes that take a long time to reduce their levels of activity. We illuminate, using three different wavelength lasers, a temporary protector (cyclododecane) and a varnish, and monitor them using a low fps rate during long-term drying. The results are compared to the traditional method. This work also presents a monitoring method that uses portable equipment. The results present the feasibility of using the portable device and show the improved sensitivity of the dynamic laser speckle when sensing the long-term process for drying cyclododecane and varnish in conservation.

## 1. Introduction

Digital holography [1,2], a well-known speckle interferometric technique, is generally used to measure paints as they dry, however, the technique demands complex experimental configurations that limit its use [3,4]. Other digital speckle pattern interferometry techniques, such as shearography, are applied for art objects, particularly canvas and panel paintings [5,6], but require a similar experimental holographical configuration. In art restoration, treatments usually change the visual aspect of the art objects. As in the case of the application of varnish in paintings, changes may appear in the brightness and light saturation [7,8,9].

The processes that provide fixation and consolidation in painting conservation use numerous substances, among which is cyclododecane (which forms bright artefacts on the surface, thus changing the final aspect of the object) [10,11,12]. One way to control the impact of art restoration is by managing the drying times: by setting the best manipulation times and so reducing surface artefacts. The timing of the evaporation of the solvents present in inks can be measured using thermogravimetric techniques [13], as well as by weighting the canvas during drying [14] (with some obvious limitations for in situ restoration).

Dynamic laser speckle (DLS), or biospeckle laser (BSL), is a non-destructive technique that monitors biological and non-biological activity. This technique can be applied on various materials with differing behaviors. Complex fluid applications include the motility of frozen bovine semen [15], blood flow [16,17], bacterial chemotaxis [18], as well as in the reaction of MEL-RC08 line cancer cells to the application of the drug Colcemid™ (Gibco BRL, Grand Island, NY, USA) [19]. In fluids, the approaches must be biased to accommodate the considerable movement produced by light scatterers within colloid or biological tissues.

The dynamics of the processes are critical in DLS, and adjustments are required in the setup, as well as in the choice of the best image processing method. In colloids, which is the case of paintings, the literature discusses DLS and paint drying [20,21,22,23], where the level of activity of the scatterers greatly varies from the moment the ink is applied, with high levels of volatilization until the end of the drying process. Thus, a method of monitoring the process without compromising the observation of the phenomenon deserves to be evaluated and tested.

The literature describes the use of commercial equipment to evaluate drying ink [24] or paint, and is presented as an alternative for use outside of optical laboratories, where the many external influences present a challenge [25] when dealing with slow paint drying processes.

Therefore, the challenge is to monitor the speckle phenomenon in situ where external influences create a barrier to many applications, and evaluate the robustness of the portable equipment [25,26]. The adjustment of the speckle analysis must also be considered, particularly for processing events that occur over a long period of time. Therefore, the installed equipment must capture the information, as well analyzing the biased information.

This work aims to test the portable equipment for monitoring the activity of treatments during painting conservation in situ by means of a modified image processing biased for long-term measurement. Two types of chemicals were tested during drying, and they were evaluated using three laser wavelengths. The portable equipment was compared to a DLS laboratorial setup, as well as weight monitoring methods.

## 2. Design and Control of Portable System

### 2.1. Structural Design

**Figure 1:**
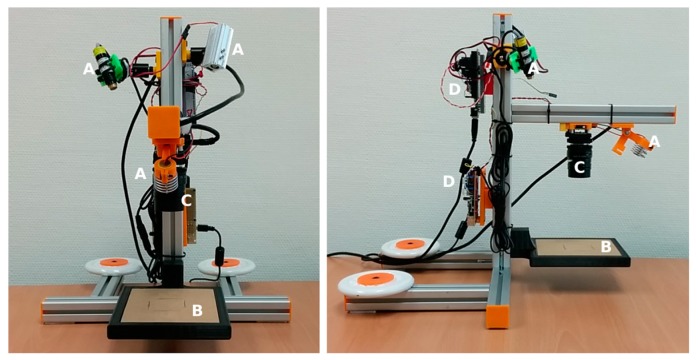
Portable experimental setup for the dynamic laser speckle. A: lasers; B: removable platform; C: camera; D: laser controller.

Figure 1 shows the elements of the portable system for dynamic speckle patterns.

It can control up to four diode-lasers, enabling rear and frontal illumination using prisms and a removable platform. The images are captured with a CCD. All the elements are assembled using aluminum T-slots. This structure is sturdy enough to capture stable images. In addition, cushion pads are glued in the base to absorb vibrations. Camera height and laser orientation can be adjusted. The B platform (Figure 1) is removable for the placement of translucent material. This enables a rear illumination setup by means of prisms and laser reorientation. As portable equipment, it can be taken to the place of the conservation and the orientation of the camera and lasers can be adjusted to monitor the horizontal and vertical disposition of the paintings.

### 2.2. Electronic Design

**Figure 2:**
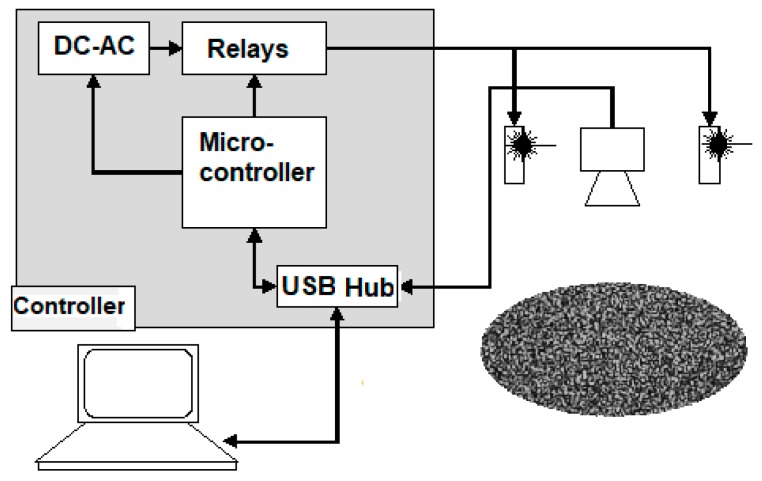
Diagram of portable system controller with the gray box representing the device control system connected to the computer, lasers, and camera.

The portable system control, as shown in Figure 2, was designed and developed at the Universitat Politècnica de València, in Spain.

**Figure 3:**
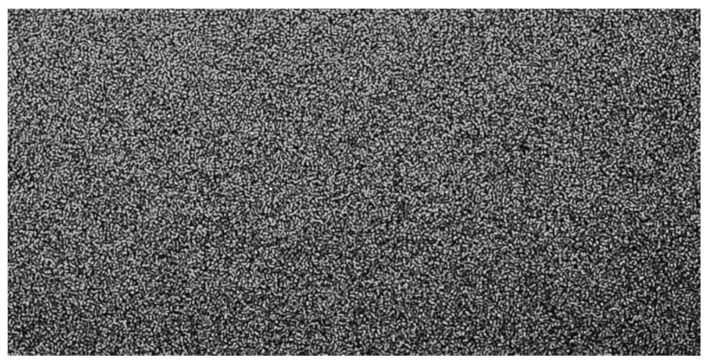
A portion of the captured speckle pattern created by the red laser diode used by the portable system (shows a real area of 4 × 2 cm).

For the experiments, three lasers were installed (infrared: 808 nm, 50 mW; red: 650 nm, 20 mW; green: 432 nm, 20 mW; from Prophotonix Limited, Salem, NH, USA). These lasers can be turned on/off using relays controlled by a microcontroller (Arduino, BCMI US LLC, Boston, MA, USA) connected to a laptop through a USB port. The camera is a USB 3.0 camera (See3Cam 2304 × 1296 pixels, 2.2 μm, no IR filter, e-con Systems Ltd, Chennai, India), also connected to a laptop. Software was developed to control image grabbing and laser synchronization. The lasers employed were not heavy-duty components; thus they can be turned on lasers on at a preselected time before capture. This reduces laser deterioration, and allow stabilization before each new capture. The software enables the periodical capture of groups of images at a specific frame rate. The capture surface can be illuminated with different lasers during the experiment. Figure 3 shows a portion of the captured speckle pattern generated on the surface by the red laser.

## 3. Materials and Methods

### 3.1. Specimen

The surface of an acrylic painted canvas was protected with two solutions:

- commercial acrylic resin varnish (Titan retouching varnish, INDUSTRIAS TITAN S.A., Barcelona, Spain, diluted in aliphatic and aromatic hydrocarbons).
- cyclododecane: saturated and chemically stable cyclic hydrocarbon (C_12_H_24_). This chemical is applied in conservation treatments on a variety of surfaces as a temporary protector. It was used as a solution of 5 g in 10 mL of ligroin (an aliphatic hydrocarbon mixture).

The process was performed by a professional painting conservator using a brush to apply homogeneous layers. Layers of a thickness of approximately 100 μm were applied considering the density of the products and the difference in weight of the canvas before/after application. The experimental conditions were 22–23 °C and 51–53% of humidity.

### 3.2. Experimental Setup Employed for Non-Portable Dynamic Laser Speckle

The experimental setup for the non-portable system consisted of a linearly polarized He-Ne laser beam (633 nm, 35 mW, Research Electro-Optics, Inc., Boulder, CO, USA). The beam size was expanded using only a microscope objective with a 10× magnification in order have a round illuminated area with 100 mm of diameter covering a 40 mm square area.

The images were acquired from a TV zoom lens with a focal length of 50 mm, numerical aperture of f/11 (speckle size was 13.57 μm), connected to an AVT Marlin F-145B CCD camera (pixel size of 4.65 μm, Allied Vision Technologies, Stadtroda, Germany) [26].

### 3.3. Protocol for Speckle Pattern Acquisition

First experiment: validation of the portable system. A collection of 64 images (8 bits, 640 × 480 pixels, and an exposure time of 1/125) were acquired using a traditional experimental system (at the beginning of each minute, during 16 min at a rate of 10 frames per second). The portable system was configured in the same way: sets of 64 images, 640 × 480, 10 fps, during 16 min. In both cases, canvases of 60 × 60 mm were painted using the cyclododecane and varnish.

Second experiment: a comparison of back and forward scattering using the portable equipment was made. The portable device has the flexibility to produce dynamic laser speckle using back and forward scattering approaches (related to the reflection and transmission of light on and through the sample respectively). The transmission can only be used when the sample allows the light to pass through the sample to the camera. A layer of cyclododecane was applied to a glass surface that was illuminated using back and forward scattering, and using the same image time rate and processing that was presented in the first experiment.

Third experiment: time rate changes and DLS with different lasers were compared to the weight monitoring method. A uniform layer was applied on the canvases. One of the canvases was placed in the dynamic laser speckle capture system and another was placed on a scale to be weighed.

The canvas was weighed using a 1 mg precision scale (GEM50 Smart Weight, Better Basics Ltd., Chestnut Ridge, NY, USA) every five minutes initially—and then every 10 min—and finally every 30 min.

The canvas was illuminated every minute by the three lasers (infrared, red, and green) alternatively, and 20 images were captured at 10 fps (frames per second). Before each image acquisition, the corresponding laser was connected for five seconds before starting the capture of images to ensure light stability. Light stability was checked for every laser using an optical power meter (OPM 842-PE, Newport Ltd., Irvine, CA, USA).

Five sets of images were created after the capture process for each laser:

- Set A: Capture of 20 images at 10 fps every minute (for fast dynamics).
- Sets B, C, D, E: Capture of {30, 15, 6, 3} images at {1 image every 1 min, 1 image every 2 min, 1 image every 5 min, 1 image every 10 min} every 30 min (for slow dynamics).

This enables measuring the drying process in the same experiment using two methods. In all cases, image quality was tested to avoid speckle grains with unneeded information about the phenomena. Therefore, the setup was biased to avoid speckle with a blurred appearance and saturated areas, and to avoid inhomogeneity in accordance with the proposed quality test protocol [27,28].

### 3.4. Methodology to Process Dynamic Speckle Images

The dynamics of the speckle variation were monitored using second-order statistics [29], building the time history speckle pattern (THSP) matrices and co-occurrence matrix (COM) using a selection of random points in the prime image to create the THSP [30]. From the COM, we obtained the absolute values of differences (AVD) method [31], expressed in Equation (1):

(1) $$AVD=\underset{ij}{\sum }COM_{ij}\left|i-j\right|$$

where the COM is the co-occurrence matrix related to the THSP, and the *i* and *j* variables represent the *i* line and the *j* column of each point of the COM matrix:

(2) $$COM=[N_{ij}]$$

The entries are the number of occurrences (N) of a certain intensity value *i* that is immediately followed by an intensity value *j*.

**Figure 4:**
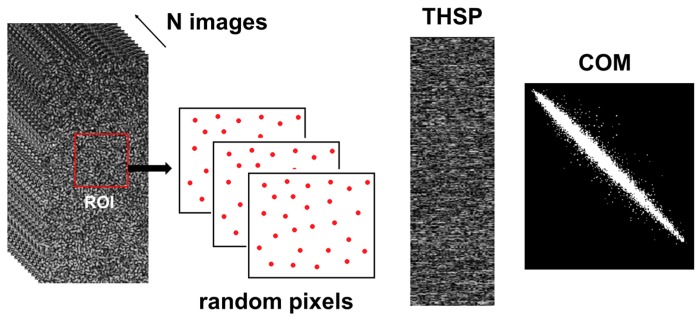
Scheme of the THSP and COM construction using random pixels.

In Figure 4, it is possible to see the THSP created—instead of using random points in the prime image in the selected ROI.

Because the pixel set to compute AVD is randomly chosen, an AVD index was computed averaging the AVD values from ten pixels sets. The error bars appearing in the figures represent the standard deviations of those values.

The AVD index is used as a measure of light scattering that can be generally associated with activity. Activity is attributed to the numerous phenomena present during the drying processes. Thus, in this context, activity should be read as solvent evaporation and other curing reactions [32] that occur during the drying process, including the adjustment of the surface.

## 4. Results

### 4.1. Validation of the Portable Device to Monitor Drying Processes in Painting Conservation

**Figure 5:**
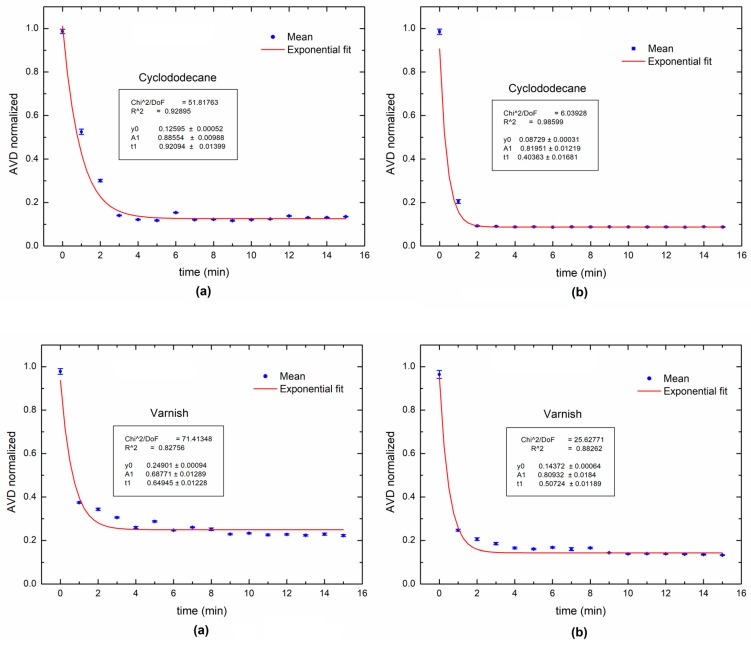
Dynamic laser speckle activity levels during the paint drying process for each painting treatments: (**a**) portable system and (**b**) lab system.

Figure 5 shows the AVD index during the varnish and cyclododecane drying process using the traditional experimental configuration and the proposed portable system. The comparison between the traditional method and the portable system was made using a characteristic drying curve, expressed by an exponential [33,34]. The fitting of the data to an exponential behavior was tested using the R^2^ index. The portable system had a lower fitting using the common method to monitor the short-term process, but still had a high R^2^ value, which revealed its reliability.

### 4.2. Comparison of Back and Forward Scattering Using the Portable Equipment

**Figure 6:**
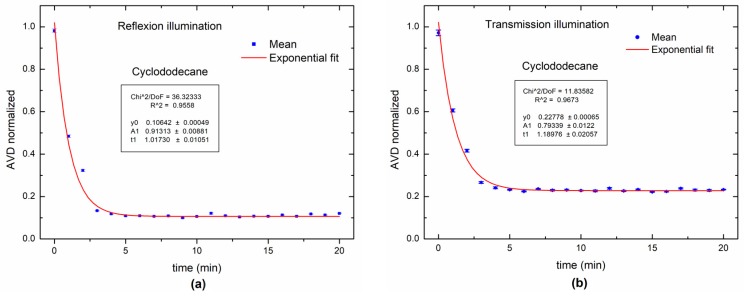
Dynamic laser speckle activity levels during paint drying process for cyclododecane: (**a**) back scattering (reflection illumination); (**b**) forward scattering (transmission illumination).

Figure 6 shows the AVD index with an exponential tendency curve when using a red laser. Two experimental configurations were used to acquire the data: back and forward scattering. In Figure 6a, the drying process of cyclododecane was sensed from 1 to approximately 0.1 in normalized values, while in Figure 6b the drying process was sensed from 1 to approximately 0.3 in normalized values.

### 4.3. Third Experiment: Changes in Time Rate Using Different Lasers Compared to Weight

**Figure 7:**
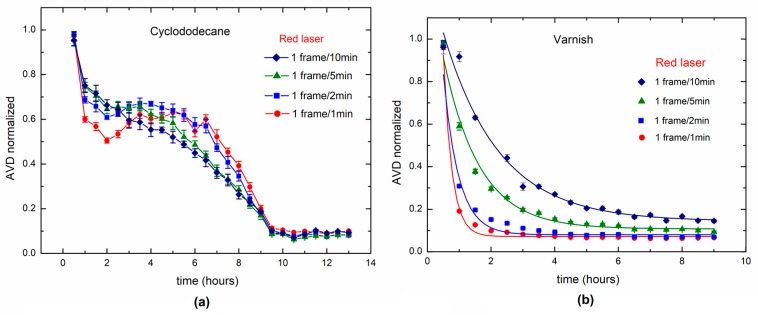
Dynamic laser speckle activity levels during the paint drying process for each painting treatment: (**a**) cyclododecane and (**b**) varnish.

**Figure 8:**
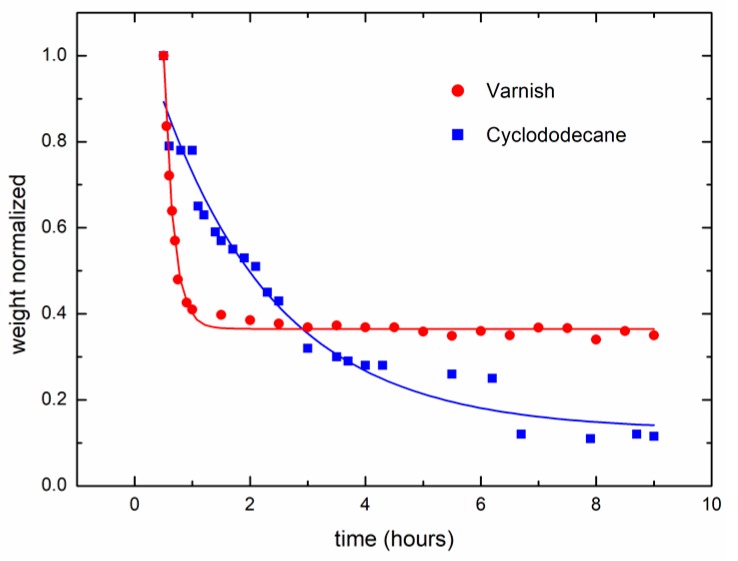
Weights of drying paint treatments over time.

The results of the proposed method to monitor long-term activities in the drying process can be seen in Figure 7, where the drying times for cyclododecane (Figure 7a) and varnish (Figure 7b) are shown. In a time-rate of 1 frame per minute, 1 frame each 2 min, 1 frame each 5 min, and 1 frame each 10 min, the drying process was monitored during 16 h. The AVD index was based on 30, 15, 6 and 3 images respectively. In Figure 7a, we can divide the curve in two parts, the first from zero to 3 h, and the second part from 8 to 16 h. The zone between 3 and 8 h can be considered as transient. In the first part, the curve behaves similarly to the drying process presented in traditional measurements of dynamic laser speckle using higher time rates (10 fps, for example, in the case of Figure 6). It is comparable with the loss of mass monitored by the scale shown in Figure 8.

However, the similarity was greater for the higher time rates, such as 1 frame per minute (1 frame/1 min) and 0.5 frame per minute (1 frame/2 min). While in the second part of the curve, the behavior is completely different from the traditional curve, shown in Figure 6, with an ability to sense small changes in the cyclododecane drying surface (where the traditional method presented a flat curve). For varnish, the behavior shown is similar to the traditional methods of drying, and this can be validated using a scale to weigh the mass loss.

Figure 8 shows the weighing process during the drying process using a scale. The speed of the drying process for the two treatments (varnish and cyclododecane) is shown. The cyclododecane shows a slower drying curve.

**Figure 9:**
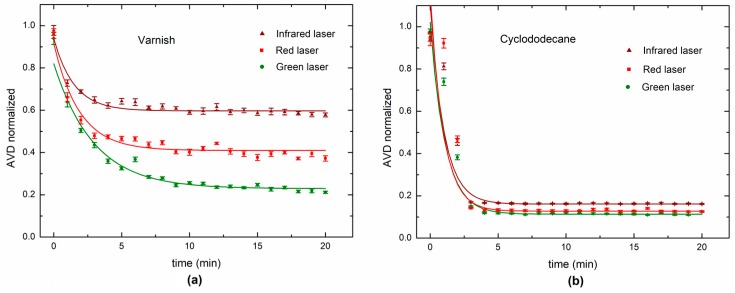
Dynamic laser speckle for fast drying dynamics for colloids using three wavelengths for each painting treatment: (**a**) varnish and (**b**) cyclododecane.

The DLS analysis with different wavelength lasers for the varnish and cyclododecane is presented in Figure 9. Normalized AVD indexes were obtained. The varnish was more sensitive to the wavelengths than the cyclododecane. An fps rate of 10 was used to acquire the images in all cases. For fast dynamics, the cyclododecane was the first colloid to dry, in contrast with the weight monitoring observation (Figure 8).

**Figure 10:**
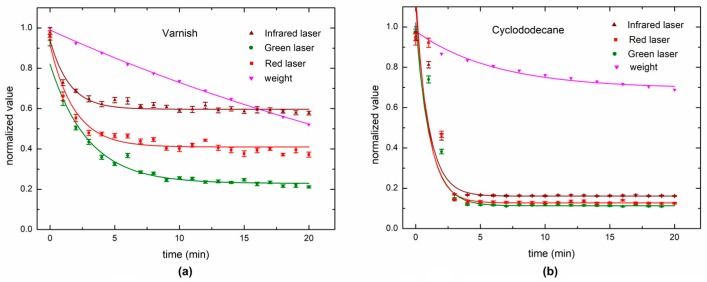
Dynamic laser speckle index for fast dynamics (10 fps) and the weighing for each painting treatment: (**a**) varnish and (**b**) cyclododecane.

Figure 10 shows the DLS and weighing values for the three wavelength lasers. Varnish and cyclododecane were monitored during 20 min, and analysed using fast dynamics, i.e., 10 fps. The fast dynamics presented the best ability to follow the fastest drying process produced by the varnish, but the DLS could not sense the cyclododecane that presented the slowest drying dynamic.

**Figure 11:**
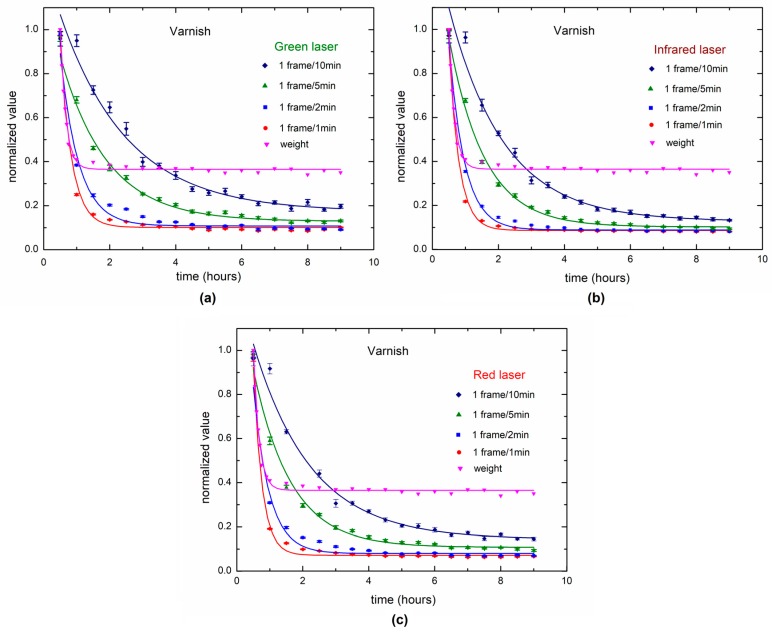
Dynamic laser speckle in low dynamics for varnish drying using the three wavelengths: (**a**) green laser (**b**) IR laser (**c**) red laser, as observed in four different time-rates and compared to the weighing.

The slow (long-term) dynamic is presented in Figure 11 for varnish drying, and the slow dynamic is also compared for weighing and the three lasers (wavelengths). Four time-rates were used to evaluate the long-term process response. The slower the time-rate, the greater is the difference in the weighing process. In this case, the acquisition of 1 frame each 10 min provided the best ability to sense changes in long-term monitoring. Otherwise, weighing output did not have the ability to follow the drying process after one hour.

The data was adjusted (Equation (3)), and the value of the variable ‘y’; ‘y_0_’; t_1_‘; and ‘A_1_‘are shown in Table 1.

(3) $$y=y_{0}+A_{1}e^{\left(\frac{-x}{t_{1}}\right)}$$

Table 1 shows the parameters of the fitting curves for low dynamics and the expected characteristic curve of the drying varnish is clearly seen.

**Figure 12:**
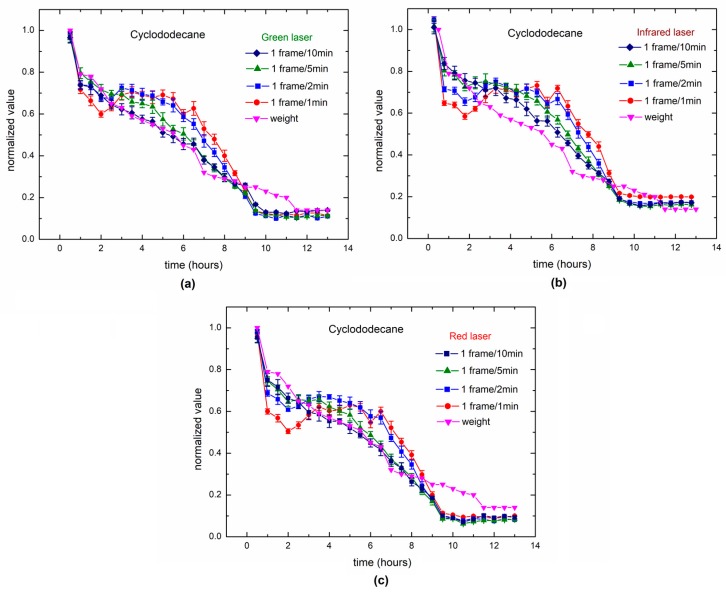
Dynamic laser speckle for drying cyclododecane with low dynamics using three wavelengths: (**a**) green laser (**b**) IR laser (**c**) red laser, observed in four different time-rates and compared to the weighed values.

In Figure 12, the result of a long-term monitoring of cyclododecane is presented in comparison to the weighing and to three lasers with different wavelengths. The ability of each time-rate to follow the process was also observed. The cyclododecane dries in the external layer first, and then the drying process continues to the inner layers. In this case, the weighing process could follow the slow drying during the hours before stabilization (which also happened with the DLS outputs using low time-rates).

## 5. Discussion

### 5.1. Validation of the Portable Device for Monitor Drying Processes during Painting Conservation

Beyond the ability of the proposed device to follow the drying process, it is relevant to highlight its ability to sense the process more smoothly than the traditional setup because of the different cameras used. In the portable device, the size of the pixel in the CCD camera was 2.2 μm, while the size of the pixel in the optical laboratory was 4.65 μm. The smoothness of the exponential curve from the proposed device enables us to follow the drying process for a longer time than when using the camera in the optical laboratory. In short, pixel size matters, and can improve the sensitivity of our sensor [26].

### 5.2. Comparison of Back and Forward Scattering Using the Portable Equipment

The ability of the back scattering to sense the drying process of cyclododecane for a wider range (from 1 to approximately 0.1 in normalized AVD index values) than forward scattering (from 1 to approximately 0.3 in normalized AVD index values) can be attributed to the lesser sensitivity of the transmission in the dynamic laser speckle outcomes [35]. This leads us to adopt the back scattering reflection as prior when possible.

### 5.3. Third Experiment: Changes in Time Rate Using Different Lasers Relative to Weight

The long-term monitoring by means of DLS revealed a better ability to follow the drying process than the weighing process. This means that over the long-term, DLS when monitored is a better sensor than the traditional DLS with high time-rate (10 fps); and also better than the weighing process that could be considered as the ‘gold standard’. The monitoring of drying paint using the traditional fps is usually restricted to the first minutes [20,23,36].

The different dynamics of drying presented by varnish and cyclododecane were better followed by the long-term methodology proposed, where for the cyclododecane, the presence of two phases of drying can explain the relation of the time-rate with fast and slow drying, each linked to the surface and inner layers of the sample.

Varying the laser wavelength can be worthwhile in fast dynamics, such it happens in biological tissues, particularly in arterial pulse [37], where a lower wavelength is more sensitive to small changes. For the varnish, the lower the wavelength, the longer the process can be sensed (Figure 11). The composition of colloid and fast drying at the surface may explain the ability of the lower wavelengths. In long-term dynamics, varying the wavelength does not show a difference in sensitiveness, but it can be useful to make the image capture adjustments in function of the color of the studied surface.

The portable equipment produced reliable results, thus offering the facility to be used in situ. The use of the IR laser (offering independence from external light) makes the equipment more robust. Commercial equipment using DLS for drying ink also uses IR [24], but is limited to fast drying dynamics and this restricts its use in long-term monitoring.

## 6. Conclusions

Portable equipment for monitoring the activity of painting treatments during the restoration of paint in situ presented reliable outcomes that were comparable with the equipment used in an optical laboratory. In addition, the portable equipment is highly configurable (different wavelength lasers, back and forward scattering illumination, automatic image capture, and laser control). With these features, the system is especially interesting when used for painting conservation (where painting treatments should be monitored in place). In other areas of knowledge, the portable equipment can be used to monitor activity in the analysis of seeds, sperm motility, fruit maturation, as well as in food evaluation tasks completed outside optical laboratories.

Modified image processing biased to a long-term measurement presented better results than the traditional method driven by fast dynamics data acquisition and analysis. The proposed long-term methodology sensed different drying dynamics. The testing of laser wavelengths proved that the most accurate measurements can be obtained in fast dynamics, and improvements can generally be obtained regarding the sample and light interaction. Weight monitoring proved to be less sensitive to long-term changes in colloids during a slow drying process, as is the case of varnish. The weight monitoring setup may not be possible in real painting conservation work, or even if it is possible, it could be more difficult to implement that the proposed DLS method.

## Figures and Tables

**Table 1:** Exponential fit for drying varnish with low dynamics using three wavelengths.

	Green Laser				Infrared Laser				Red Laser			
	y_0_	A_1_	t_1_	%	y_0_	A_1_	t_1_	%	y_0_	A_1_	t_1_	%
1 image/1 min	0.10	0.84	0.35	91.5	0.09	0.83	0.31	94.0	0.07	0.85	0.53	89.6
1 image/2 min	0.11	0.79	0.55	93.6	0.09	0.85	0.47	97.4	0.08	0.75	0.96	91.6
1 image/5 min	0.13	0.75	1.41	98.4	0.10	0.86	1.07	98.9	0.11	0.80	2.26	97.7
1 image/10 min	0.17	0.90	2.04	98.2	0.13	0.97	1.67	98.7	0.14	0.89	3.50	98.7
